# Gonadotropin-releasing hormone agonist for ovulation trigger – OHSS prevention and use of modified luteal phase support for fresh embryo transfer

**DOI:** 10.1080/03009734.2020.1736696

**Published:** 2020-05-04

**Authors:** Juan Carlos Castillo, Thor Haahr, María Martínez-Moya, Peter Humaidan

**Affiliations:** a Instituto Bernabeu, Alicante, Spain;; b Department of Clinical Medicine, Aarhus University, Aarhus, Denmark;; c The Fertility Clinic Skive, Skive Regional Hospital, Skive, Denmark

**Keywords:** GnRHa trigger, hCG, IVF, ovarian hyperstimulation syndrome, ovulation trigger

## Abstract

The introduction of gonadotrophin-releasing hormone agonist (GnRHa) trigger greatly impacted modern IVF treatment. Patients at low risk of ovarian hyperstimulation syndrome (OHSS) development, undergoing fresh embryo transfer and GnRHa trigger can be offered a virtually OHSS-free treatment with non-inferior reproductive outcomes by using a modified luteal phase support in terms of small boluses of human chorionic gonadotrophin (hCG), daily recombinant luteinizing hormone LH (rLH) or GnRHa. In the OHSS risk patient, GnRHa trigger can safely be performed, followed by a ‘freeze-all’ policy with a minimal risk of OHSS development and high live birth rates in the subsequent frozen embryo transfer cycle. Importantly, GnRHa trigger opened the ‘black box’ of the luteal phase, promoting research in the most optimal steroid levels during the luteal phase. GnRHa trigger allows high-dose gonadotropin stimulation to achieve the optimal number of oocytes and embryos needed to ensure the highest chance of live birth. This review thoroughly discusses how the GnRHa trigger concept adds safety and efficacy to modern IVF in terms of OHSS prevention. Furthermore, the optimal luteal phase management after GnRHa trigger in fresh embryo transfer cycles is discussed.

## Introduction

Traditionally a bolus of human chorionic gonadotrophin (hCG) has been the gold standard for ovulation induction and final oocyte maturation in assisted reproductive technology (ART) cycles as a surrogate for the natural mid-cycle luteinizing hormone (LH) surge. However, the increased glycosylation of hCG results in a prolonged bioactivity which in combination with the sustained luteotropic activity may induce ovarian hyperstimulation syndrome (OHSS), one of the most serious complications of controlled ovarian stimulation (COS) (1). Additionally, studies have reported adverse effects of hCG in terms of reduced endometrial receptivity and a negative impact on oocyte as well as embryo quality (2–4).

**Figure 1.**
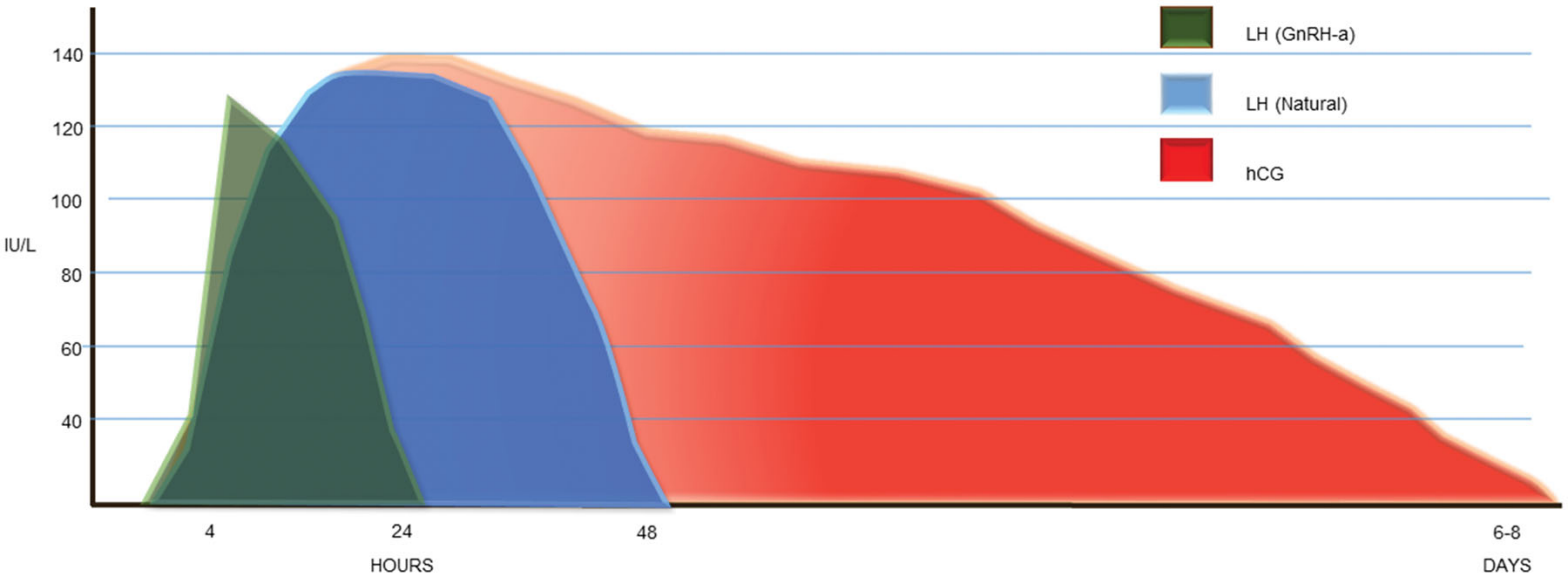
Comparison of ovulation triggers. Schematic graphic showing LH activity of different types of trigger agents when compared with natural mid-cycle surge. GnRHa: gonadotrophin-releasing hormone agonist; hCG: human chorionic gonadotropin. Adapted from Hoff et al. (6) and Casper (7).

When gonadotrophin-releasing hormone (GnRH) antagonist protocols were introduced for the prevention of a premature LH surge (5) it became possible to trigger final oocyte maturation and ovulation with a single bolus of a GnRH agonist (GnRHa) as an alternative to hCG. Induction of final oocyte maturation with a bolus of GnRHa in patients undergoing ovarian stimulation for IVF could be considered to be more physiologic because the elicited surge mimics the natural cycle surge of gonadotropins (Figure 1). However, when compared to the natural cycle, the total amount of gonadotrophins released during the GnRHa-triggered surge is significantly reduced, but a possible advantage of GnRHa trigger over hCG trigger is the simultaneous induction of an FSH (follicle-stimulating hormone) surge (8). The exact role of the mid-cycle FSH surge in the natural cycle is not fully understood, but available evidence suggests that FSH promotes oocyte nuclear maturation, i.e. resumption of meiosis (9,10), cumulus expansion, and the induction of LH receptors on granulosa cells to promote corpus luteum (CL) formation (11,12). With this in mind, a bolus of GnRHa induces a more physiologic final oocyte maturation; however, this comes at the expense of a deficient luteal phase due to the short duration of the induced LH/FSH peak (Figure 1). It also prevents the secretion of vasoactive substances, mainly VEGF (vascular endothelial growth factor), from the corpora lutea (13–15), thereby acting as a luteolytic agent. Luteolysis induced by GnRHa is the key to prevention of OHSS (16).

## Avoiding OHSS in risk patients with the use of GnRHa trigger

The syndrome OHSS was already described in 1941 (17). In spontaneous pregnancy, OHSS is an extremely rare event (18). Hence, OHSS is an iatrogenic complication in almost all cases and occurs almost exclusively in the presence of sustained LH activity as seen after hCG trigger in ART cycles. Furthermore, the severity of OHSS is proportional to the dose of hCG administered and the number of corpora lutea obtained after trigger. OHSS occurs less frequently after mild ovarian stimulation using clomiphene citrate or low-dose gonadotrophins for the development of a few follicles in intrauterine insemination (IUI) cycles (18,19). It has, however, previously been reported to be as high as 30% in polycystic ovarian syndrome (PCOS) patients submitted to IVF (13).

The most significant benefit of GnRHa trigger is its ability to induce a quick and reversible luteolysis and, thus, reduce the risk of OHSS development. In GnRH antagonist cycles, GnRHa trigger has been utilized to induce the final stage of follicular maturation while reducing the risk of OHSS. This protocol works because GnRH agonists have a greater affinity for the GnRH receptor than GnRH antagonists have. Thus, a bolus of GnRHa can displace the GnRH antagonist from the receptor, resulting in a surge of LH as well as FSH. The lower mean LH concentrations and decreased LH pulse amplitude and activity over the luteinized granulosa/theca cells in addition to an upregulation of the apoptosis of granulosa cells induce the process of luteolysis early in the luteal phase, which seems to be key factors for OHSS prevention after GnRHa trigger (20,21). Interestingly, in a small pilot study, it was reported that converting OHSS risk patients during stimulation from a long GnRHa protocol to a GnRH antagonist protocol and subsequent GnRHa trigger is a plausible rescue option in patients at high risk of OHSS development (22). Currently, however, as the benefits and risks of this rescue strategy are not quite clear, most clinicians probably opt for cycle segmentation and frozen–thawed embryo transfer in a subsequent cycle.

From a clinical point of view, the most optimal strategy to prevent OHSS starts with the identification of OHSS risk patients in order to adapt the ovarian stimulation regimen accordingly and subsequently plan the trigger agent. In patients at risk of OHSS, some clinicians might opt for elective frozen embryo transfer (eFET). It has, however, been proven, during a series of randomized controlled trials (RCTs), that GnRHa trigger enables a fresh transfer if modifications of the luteal phase support (LPS) are performed, and, importantly, the reproductive outcome is non-inferior to hCG trigger (4,23–26). However, only GnRHa trigger and eFET (segmentation) will result in a virtually ‘OHSS-free clinic’ (27). Nevertheless, on rare occasions, OHSS can be encountered even when using GnRHa trigger and eFET (28).

## GnRHa trigger and segmentation (freeze-all)

Oocyte/embryo freezing, so-called ‘freeze-all’, segmentation or eFET, was recently proposed for all GnRHa-triggered IVF cycles (27,29) and in particular so for women at risk of OHSS (30), avoiding early as well as late-onset OHSS. The segmented treatment approach involves: (1) controlled ovarian hyperstimulation (COH) using a GnRHa trigger; (2) vitrification of oocytes/embryos; and (3) embryo transfer in a subsequent, natural or artificial cycle. By segmenting the treatment into these steps, the concern about specific and adequate luteal support after GnRHa triggering disappears. An additional benefit of postponing embryo transfer is avoiding embryo exposure to non-physiologic, elevated circulating steroid levels observed in fresh transfer IVF cycles. In recent years, the pregnancy and live birth rates after the transfer of frozen embryos have improved, most certainly as a result of the use of vitrification for embryo cryopreservation (31).

A recent meta-analysis comparing fresh transfer to FET supports the use of eFET in hyper-responder patients and patients undergoing PGT-A (preimplantation genetic testing for aneuploidy) (32); however, from a clinical point of view, it is warranted to make a study comparing reproductive outcomes in fresh and eFET cycles triggered with GnRHa in polycystic ovary (PCO) patients, who are at high risk of OHSS. Unfortunately, such a study is not available, and results of a multicenter, randomized clinical trial comparing fresh transfer with eFET in hCG-triggered cycles among infertile women with PCOS showed that eFET was associated with a higher live birth rate than fresh embryo transfer (49.3% versus 42.0%). This difference was explained by a lower early pregnancy loss rate in the eFET group (22.0% versus 32.7%). However, it is also worth highlighting that a higher incidence of preeclampsia was seen in the eFET group compared with the fresh transfer group (4.4% versus 1.4%) (33). Thus, some concerns and drawbacks regarding the freeze-all approach need to be weighed against the potential benefits. A frozen cycle involves higher economical costs and additional emotional/physical burden associated with deferring the programmed FET, and the additional endometrial preparation protocol (ultrasound controls, medication, office visits). Importantly, additional manipulations (freezing/thawing) at early embryonic stages might induce epigenetic changes only visible during adulthood (34), and the oestrogen contents of the artificial cycle might have an impact on placentation. Finally, despite the freeze-all policy clearly preventing the development of late-onset OHSS, it does not completely prevent severe early OHSS, as recently reported (35).

Although there is a lack of randomized clinical trials comparing GnRHa-triggered fresh and eFET cycles, evidence from observational studies suggests that GnRHa triggering combined with oocyte/embryo vitrification and embryo transfer in a subsequent cycle provides patients at high risk of developing OHSS a safe and efficient alternative. In this high-risk population, a freeze-only approach to minimize the risk of OHSS may outweigh the known and unknown risks and the financial costs involved with the eFET strategy. Nonetheless, results from large long-term follow-up trials in children conceived as a result of frozen–thawed embryo transfer and the exploration of possible obstetric complications are mandatory before the widespread adoption of a ‘freeze-all ‘policy can be recommended.

## GnRH trigger and the optimal modified luteal phase support (mLPS) during fresh embryo transfer

Luteal phase insufficiency, resulting in an unacceptably high early pregnancy loss rate, despite the use of a standard LPS was seen during the early studies of GnRHa trigger (36,37). Following these first disappointing reports, several studies explored how to most optimally rescue the luteal phase after GnRHa trigger (8). Current evidence supports that GnRHa trigger and fresh transfer followed by a modified LPS (mLPS) policy results in reproductive outcomes comparable to those of hCG trigger – and notably with a reduction in OHSS (4). This introduced the idea of individually tailoring the luteal phase after GnRHa trigger according to the response to ovarian stimulation and luteal steroid levels (25,38–40).

### mLPS: a single low-dose bolus of hCG

As noted previously, while the lower LH activity commencing early in the luteal phase was a beneficial key factor for OHSS prevention, it was also responsible for the disappointing lower reproductive outcomes in GnRHa-triggered compared with hCG-triggered cycles. Thus, the question was: how can LH activity be introduced after GnRHa triggering to most optimally restore the luteal phase?

Based on two small pilot studies in IUI patients (41,42), the concept of an early LPS with a single low-dose hCG (1500 IU) on the day of ovum pick-up (OPU) combined with a standard LPS (vaginal progesterone and oral oestradiol) was investigated (38). The small bolus of hCG clearly rescued the luteal phase after GnRHa triggering, resulting in a satisfying reproductive outcome. The protocol was subsequently investigated in a large RCT showing a non-significant difference in delivery rates between GnRHa-triggered cycles supplemented with a single bolus of hCG versus 10,000 IU hCG for trigger (24). Importantly, no OHSS was found in the GnRHa group versus 2% in the 10,000 IU hCG group, even though more than one-third of the patients in each group had ≥14 follicles ≥11 mm on the day of triggering. To further explore whether this procedure was safe for women at risk of OHSS, a total of 118 patients at risk of OHSS were randomized to either hCG trigger or the modified GnRHa trigger protocol (25). Of note, women with more than 25 follicles were excluded from the trial. No OHSS was reported after GnRHa trigger using this upper follicular limit compared with 3% of women experiencing OHSS after hCG trigger. Additional retrospective studies also explored the use of the same protocol in at-risk patients, resulting in similar outcomes: high pregnancy rates and a very low OHSS incidence (0.72%−1.4%). It is worthy of note, however, that these retrospective analyses included cycles with no upper limit in terms of number of follicles present on the trigger day (43,44). In contrast, one retrospective cohort study included 23 women at high risk of OHSS development who underwent GnRHa trigger and also received a bolus of 1500 hCG on the day of oocyte retrieval as previously reported (24). In this OHSS high-risk group, the severe OHSS risk was reported to be high, 26% (6/23), which seems conceivable as no upper limit of follicles for fresh transfer at trigger was applied and as many as 50–65 oocytes were retrieved in some patients (45). A recent trial investigated the advantage of adding GnRHa (triptorelin 0.1 mg s.c.) on day 6 after OPU (OPU + 6) in an RCT compared with GnRHa trigger and modified LPS with 1500 IU hCG at OPU (46). The intervention with GnRHa at OPU + 6 showed a small but non-significant increase in live birth rates (OR 1.3; 95% CI 0.8–2.0).

Based on available evidence, GnRHa trigger followed by a single low-dose bolus of hCG and fresh transfer prevents or significantly reduces the risk of OHSS while providing high pregnancy rates. However, it is crucial to have an upper cut-off limit of follicles before embarking on this strategy. Based on our previous studies and daily clinical experience we suggest a cut-off of >25 follicles ≥11 mm (47).

### mLPS: recombinant LH (rLH)

Theoretically, the addition of recombinant LH (rLH) will overcome the defective luteal endocrine/endometrial environment and restore luteal function after GnRHa trigger. Due to the shorter half-life of rLH compared with hCG, the risk of OHSS may be further reduced. Papanikolaou et al. explored this interesting concept in a pilot study, applying repeated doses of rLH as a luteal supplementation (six alternate doses of 300 IU rLH, starting on the day of oocyte retrieval in addition to vaginally administered micronized progesterone) post GnRHa trigger and compared this concept to a standard hCG trigger and LPS (48). Similar implantation rates were achieved with the rLH luteal supplementation scheme compared with the standard luteal progesterone protocol (25.0% versus 26.7%, respectively). No cases of OHSS were noticed in this group of normal-responder patients. Thus, studies exploring rLH supplementation in patients at risk of OHSS are lacking. At present, more extensive studies are warranted to draw robust conclusions regarding the cost/efficacy and dose of rLH for the most optimal luteal support after GnRHa trigger, specifically in the at-risk population. It is noteworthy that the cost of rLH and patient convenience might hinder the use of rLH in a standard luteal support practice.

### mLPS: exogenous intensive progesterone and oestradiol supplementation

An early small study in OHSS risk patients by Babayof et al. (13) explored the concept of steroids-only luteal phase supplementation after GnRHa trigger and also investigated markers of luteal phase function in order to identify patients in need of intense support after GnRHa trigger. More recently, the approach was investigated in a RCT, comparing GnRHa trigger to hCG trigger, and using intensive LPS and monitoring of serum steroid levels in the GnRHa-triggered group (49). All patients received 50 mg intramuscular progesterone daily starting the evening after oocyte retrieval and continued until 10 weeks of gestation. In addition, patients received three 0.1-mg oestradiol (E2) transdermal patches every other day starting on the day after oocyte retrieval. Serum E2 and progesterone were measured on the day of embryo transfer, 1 week after oocyte retrieval, and weekly thereafter. The dose of transdermal E2 patches was increased, if necessary, to a maximum of four 0.1-mg patches every other day, and/or the addition of oral micronized E2 to maintain a serum E2 level >200 pg/mL. The intramuscular progesterone dose was also increased, if necessary, to a maximum of 75 mg daily and/or the addition of micronized vaginal progesterone to maintain a serum progesterone level >20 ng/mL.

An ongoing pregnancy rate of 53% and no OHSS was reported in the GnRHa trigger group compared with 34% (10/29) in the hCG trigger group. Some groups, applying a similar exogenous intensive luteal support regimen, reported favourable results after fresh transfer in retrospective studies (44,50). However, others were not able to corroborate the initial findings (51). Finally, the use of daily intramuscular injections of progesterone for up to 10 weeks and the perceived reduced patient-friendliness of this LPS policy is a potential drawback for the widespread use of this approach in clinical practice.

Despite the potential limitations, but considering the encouraging results, the Engmann group evaluated the factors predicting the probability of successful outcomes after GnRHa trigger, reporting that a peak serum E2 ≥ 4000 pg/mL and serum LH levels on the day of trigger are the most important predictive factors for success (52). The implantation (34.4% versus 25.3%; *p* = 0.02) and clinical pregnancy rates (53.6% versus 38.1%; *p* = 0.02) were significantly higher in patients with peak E2 levels ≥4000 pg/mL compared with those with peak E2 levels <4000 pg/mL. Patients within these limits might also have higher LH levels during the luteal phase, leading to a hypothetically higher degree of CL rescue, higher progesterone and E levels, and subsequently higher implantation rates. The authors hypothesized that some form of low-dose LH-like activity might rescue CL function in patients with low peak serum E2 levels without significantly increasing the risk of OHSS. Following this, the authors suggested administering low-dose adjuvant hCG in women with peak serum E2 < 4000 pg/mL, in line with the proposed protocol by Humaidan et al. (23–25,38).

### mLPS: the dual trigger concept – concomitant use of GnRHa and low-dose hCG

Shapiro et al. first reported the use of the so-called dual trigger (GnRHa plus low-dose hCG) (53). In their retrospective analysis, patients with significant risk factors for OHSS received a combination of 4 mg leuprolide acetate and hCG in a dose ranging from 1000 IU to 2500 IU for trigger, depending on body weight (mean dose 26.2 IU hCG/kg) and follicle number >25. The authors reported good pregnancy rates and no OHSS cases, using this protocol. Of note, this trial did not include a control group for comparison. In a subsequent larger study in a total of 182 women at risk of OHSS who had individual dosing of the hCG bolus used for dual trigger (54), the same group reported that concomitant low-dose hCG with a GnRHa trigger was associated with higher ongoing pregnancy and implantation rates and reduced pregnancy loss rates when compared with GnRHa trigger only. Moreover, a low OHSS rate of 0.5% was reported.

Another retrospective analysis including a total of 102 IVF/ICSI (intracytoplasmic sperm injection) cycles in OHSS high-risk patients having peak serum E2 levels <4000 pg/mL reported that the use of dual trigger (GnRHa and a fixed dose of 1000 IU hCG) resulted in a significantly higher live birth rate (52.9% versus 30.9%), implantation rate (41.9% versus 22.1%), and clinical pregnancy rate (58.8% versus 36.8%) compared with GnRHa trigger only (55). In both groups, an intensive LPS was used, consisting of 50 mg intramuscular progesterone daily and 0.3 mg transdermal E patches every other day, starting from the day after OPU. Additional oral E and intramuscular progesterone were used up to 10 weeks of gestation, maintaining levels above 200 pg/mL and 20 ng/mL, respectively. One mild OHSS case was reported in the dual trigger group, whereas there was no OHSS in the GnRHa trigger group. The authors concluded that the dual trigger concept using a combination of GnRHa and low-dose hCG in high responders with peak E2 < 4000 pg/mL improved live birth rates without increasing the risk of clinically significant OHSS.

Taken together, data from observational analyses support the idea, that, taking into account oestradiol levels on the trigger day and luteal function markers, monitoring with appropriate dose adjustment seems important when deciding whether dual trigger or steroids only should be used to support the luteal phase of patients at risk of OHSS development. This is in the absence of RCTs evaluating the optimal timing and dosage of hCG to be given alongside GnRHa trigger.

### mLPS: intranasal gonadotropin-releasing hormone agonist

The idea of only supporting the luteal phase after GnRHa with daily doses of intranasal GnRHa was initially explored in 17 and 40 patients, respectively (56,57). More recently, the concept was tested in a retrospective study, including 46 OHSS high-risk patients, using a daily intranasal dose of GnRHa for LPS in GnRHa-triggered cycles. Importantly, no exogenous steroids were used for LPS (58). Of the 46 patients analysed, 52.1% achieved an ongoing pregnancy. The treatment was well tolerated by all patients, and none of the patients developed clinical signs of early or late OHSS.

The same group subsequently retrospectively reported the outcomes of 1436 cycles triggered with hCG, using repeated daily nasal GnRHa administration as the sole LPS. Higher live birth rates were reported with this protocol when compared with a retrospective cohort of 1093 patients having daily vaginal LPS after hCG trigger (59). The potential advantages of this approach over current strategies are: (i) convenient nasal self-administration compared with hCG injections or multiple daily vaginal administrations of progesterone; (ii) use of a purely synthetic polypeptide with no biological fluid residues; (iii) the opportunity for early pregnancy diagnosing (56). Larger prospective trials are clearly needed to draw firm conclusions regarding the true benefits of the use of intranasal GnRHa for LPS.

## Conclusions

The introduction of the GnRHa trigger in modern IVF revolutionized ovarian stimulation and ovarian trigger concepts. Moreover, it opened the ‘black box’ of the luteal phase. Currently, patients undergoing fresh embryo transfer who are not at risk of OHSS development can be offered a virtually OHSS-free treatment in addition to good reproductive outcomes with modifications of the LPS after GnRHa trigger in terms of small boluses of hCG, daily rLH, or GnRHa. Although recent RCTs investigate further fine-tuning of the modified LPS, more studies are needed to reach a consensus on the optimal strategy. In the OHSS risk patient GnRHa trigger can be safely performed, followed by a ‘freeze-all’ policy with a minimal risk of OHSS development and high live birth rates in the subsequent FET cycle. Taken together, the GnRHa trigger concept adds safety, efficacy, and patient-friendliness to modern IVF.
